# A meta-analysis including dose-response relationship between night shift work and the risk of colorectal cancer

**DOI:** 10.18632/oncotarget.4502

**Published:** 2015-07-10

**Authors:** Xiao Wang, Alin Ji, Yi Zhu, Zhen Liang, Jian Wu, Shiqi Li, Shuai Meng, Xiangyi Zheng, Liping Xie

**Affiliations:** ^1^ Department of Urology, The First Affiliated Hospital, School of Medicine, Zhejiang University, Hangzhou 310003, Zhejiang Province, People's Republic of China

**Keywords:** night shift work, colorectal cancer, meta-analysis, epidemiology, risk factor

## Abstract

A meta-analysis was conducted to quantitatively evaluate the correlation between night shift work and the risk of colorectal cancer. We searched for publications up to March 2015 using PubMed, Web of Science, Cochrane Library, EMBASE and the Chinese National Knowledge Infrastructure databases, and the references of the retrieved articles and relevant reviews were also checked. OR and 95% CI were used to assess the degree of the correlation between night shift work and risk of colorectal cancer via fixed- or random-effect models. A dose-response meta-analysis was performed as well. The pooled OR estimates of the included studies illustrated that night shift work was correlated with an increased risk of colorectal cancer (OR = 1.318, 95% CI 1.121–1.551). No evidence of publication bias was detected. In the dose-response analysis, the rate of colorectal cancer increased by 11% for every 5 years increased in night shift work (OR = 1.11, 95% CI 1.03–1.20). In conclusion, this meta-analysis indicated that night shift work was associated with an increased risk of colorectal cancer. Further researches should be conducted to confirm our findings and clarify the potential biological mechanisms.

## INTRODUCTION

Colorectal cancer, which includes colon cancer and rectal cancer, is the third most common cancer in men and the second most common in women worldwide [1]. It occupies a great proportion of the global burden of cancer morbidity and mortality [1]. In the past decades, colorectal cancer morbidity increased significantly in developing countries where lifestyles changed drastically [2]. Previous studies have illustrated that some kind of lifestyles could result in different types of cancer [3–6]. These lifestyle risk factors, together with genetic susceptibilities, could not explain all the possible reasons for colorectal cancer risk in the population, and the majority proportion of colorectal cancer etiology is still poorly elucidated [7, 8].

Shift work is an important part of some kind of works including transportation, healthcare, and public service. In 2004–2005, the prevalence of shift work was 12.4% among the US female working population and 17.4% in European countries, while the trend was increasing rapidly [9]. Recently, shift work attracted increasing attentions from the public regarding to its potential role in carcinogenesis. In 2007, the IARC (International Agency for Research on Cancer) considered ‘shift work that involves circadian disruption’ to be probably carcinogenic (Group 2A) on account of powerful evidence from experimental researches but limited evidence from epidemiological studies [10].

However, previous studies investigating the correlation between night shift work and risk of colorectal cancer have illustrated conflicting findings. Some studies have demonstrated a significant correlation [11–13], others have failed to demonstrate any significant correlation [14–16]. Meta-analysis is an important tool for illustrating trends that may not be apparent in a single research. Summarizing independent studies could increase the confidence in the results [17]. To the best of our knowledge, no meta-analysis regarding the correlation between night shift work and risk of colorectal cancer has been published prior to this study. The purpose of this study was to quantitatively evaluate the correlation between night shift work and risk of colorectal cancer by summarizing the results of published case-control and cohort studies. We further assessed the correlation by using a dose-response meta-analysis approach.

## RESULTS

### Description of the meta-analysis

**Figure 1:**
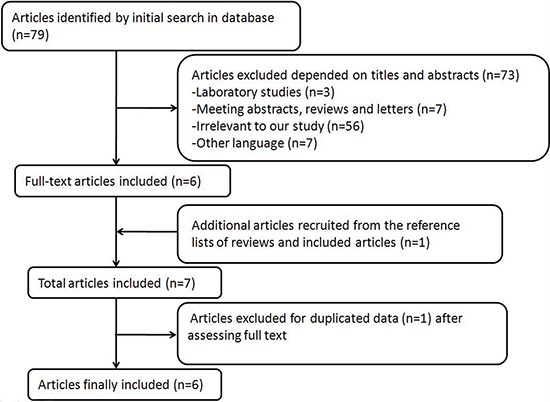
Process of article selection

Figure 1 demonstrates the detailed process of articles identification and selection. Finally, a total of 6 articles evaluating the correlation between night shift work and risk of colorectal cancer between 1996 and 2014 were included. Articles including different type of colorectal cancer and genders were considered independent researches.

Among the included articles, 3 were cohort studies [14–16] and 3 case-control studies [11–13]. Three researches were conducted in America [12, 13, 15], and 3 in Europe [11, 14, 16]. Four studies included colon cancer [13–16], and 4 included rectal cancer [13–16]. Two studies included a male population [13, 14] and 4 included a female population [12, 14–16]. Five studies adjusted for > 3 confounders [11–15] and 2 studies for ≤ 3 confounders [13, 16]. Information was collected from interview, questionnaire or database. The study quality scores, evaluated by the NOS, ranged from 5 to 9 (with a mean of 7.3). The characteristics of the qualified studies were listed in Table 1.

**Table 1:** Characteristics of published cohort and case-control studies on night work shift and risk of colorectal cancer

First Author	Published year	No. of cases/No. of subjects	Study design	Quality score	Region	Type of Cancer	gender	Range of night work shift	Variables of adjustment	Expossure assessment
Papantonion	2014	1658/5046	case-control	5	Spain	colorectal cancer	both	never vs. >30 years	Lifetime occupational history on daily time schedule of each job, day/night/rotating shifts, light at night exposure, and duration of different job	interview
Tsai	2013	1412/2176	case-control	8	US	colorectal cancer	female	daytime vs. regular night shift	obesity, smoking status, alcohol consumption, race, income, education, health insurance coverage, and marital status	interview
Parent	2012	439/951	case-control	9	Canada	colon cancer	male	never vs. ever	Smoking, BMI, alcohol, β-carotene, occupational, physical activity	interview
Parent	2012	236/748	case-control	9	Canada	rectal cancer	male	never vs. ever	Smoking, beer, BMI	interview
Schwartzbaum	2007	449/2102126	cohort	8	Sweden	colon cancer	male	never vs. ever	age, socioeconomic status, occupational position, and county of residence.	interview
Schwartzbaum	2007	326/2102126	cohort	8	Sweden	rectal cancer	male	never vs. ever	age, socioeconomic status, occupational position, and county of residence.	interview
Schwartzbaum	2007	16/1148661	cohort	8	Sweden	colon cancer	female	never vs. ever	age, socioeconomic status, occupational position, and county of residence.	interview
Schwartzbaum	2007	4/1148661	cohort	8	Sweden	rectal cancer	female	never vs. ever	age, socioeconomic status, occupational position, and county of residence.	interview
Schernhammer	2003	347/78586	cohort	8	US	colon cancer	female	never vs. >15 years	age in years, pack-years of smoking before age 30 in quintiles, body mass index in five categories, physical activity in quintiles, regular aspirin use, screening endoscopy during the study period, consumption of beef, pork, or lamb as a main dish, alcohol consumption status, total caloric intake in quintiles, use of postmenopausal hormones, menopausal status, and height in seven categories	questionnaire
Schernhammer	2003	103/78586	cohort	8	US	rectal cancer	female	never vs. >15 years	age in years, pack-years of smoking before age 30 in quintiles, body mass index in five categories, physical activity in quintiles, regular aspirin use, screening endoscopy during the study period, consumption of beef, pork, or lamb as a main dish, alcohol consumption status, total age in years, pack-years of smoking before age 30 in quintiles, body mass index in five categories, physical activity in quintiles, regular aspirin use, screening endoscopy during the study period, consumption of beef, pork, or lamb as a main dish, alcohol consumption status, total caloric intake in quintiles, use of postmenopausal hormones, menopausal status, and height in seven categories	questionnaire
Schernhammer	2003	602/78586	cohort	8	US	colorectal cancer	female	never vs. >15 years	age in years, pack-years of smoking before age 30 in quintiles, body mass index in five categories, physical activity in quintiles, regular aspirin use, screening endoscopy during the study period, consumption of beef, pork, or lamb as a main dish, alcohol consumption status, total caloric intake in quintiles, use of postmenopausal hormones, menopausal status, and height in seven categories	questionnaire
Tynes	1996	9/2619	cohort	6	Norway	colon cancer	female	never vs. ever	shift work and duration of employment	database
Tynes	1996	6/2619	cohort	6	Norway	rectal cancer	female	never vs. ever	shift work and duration of employment	database

### Risk assessment

**Figure 2:**
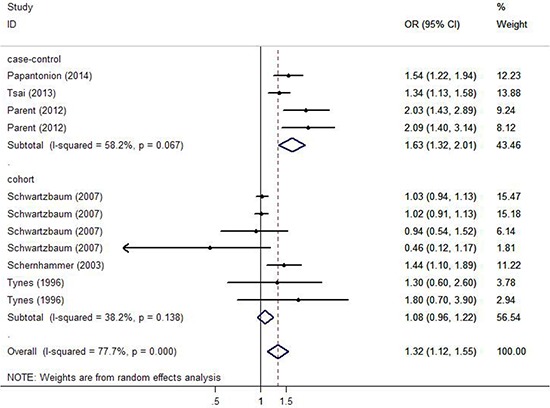
Forest plots depicting the risk estimates from included studies on the association between night shift work and risk of colorectal cancer

Figure 2 demonstrates the multivariable-adjusted ORs of each study and the combination of all studies for the longest versus shortest period of night shift work. The pooled OR indicated that night shift work was correlated with an increased risk of colorectal cancer (OR = 1.318, 95% CI 1.121–1.551). However, a statistically significant heterogeneity was found (I^2^ = 77.7%, *p* < 0.01). Subsequently, the pooled ORs grouped by study design were also calculated. We detected similar result in the case-control group as well (OR = 1.630, 95% CI 1.324–2.007), and a significant heterogeneity could not be avoided (I^2^ = 58.2%, *p* = 0.067). On the contrary, no such correlation was found in the cohort group (OR = 1.318, 95% CI 0.957–1.219), with a moderate heterogeneity (I^2^ = 38.2%, *p* = 0.138).

In the analysis stratified by the specific type of colorectal cancer, night shift work was potentially associated with an increased risk of colon cancer and rectal cancer with a significant heterogeneity ( *p* < 0.01). However, no significant statistically difference was observed (OR = 1.281, 95% CI 0.949–1.733; OR = 1.318, 95% CI 0.814–2.064, respectively). In the analysis stratified by gender, a significant positive correlation between night shift work and risk of colorectal cancer was observed in both female and male groups (OR = 1.303, 95% CI 1.100–1.544; OR = 1.328, 95% CI 1.039–1.697, respectively). In the analysis stratified by district, night shift work illustrated a significant carcinogenic effect on colorectal cancer in the America (OR = 1.610, 95% CI 1.293–2.006), but no such effect was observed in Europe (OR = 1.120, 95% CI 0.959–1.307). In the analysis stratified by exposure assessment, a more significant correlation was observed in the self-administered questionnaire group (OR = 1.440, 95% CI 1.100–1.890) compared with the interview group (OR = 1.290, 95% CI 1.073–1.551). Nevertheless, no such correlation was found in the database group (OR = 1.491, 95% CI 0.854–2.604). In the analysis stratified by control factors, a statistically significant correlation was found between night shift work and increased risk of colorectal cancer in both the group adjusted for >3 control factors (OR = 1.248, 95% CI 1.058–1.472) and the group adjusted for ≤ 3 control factors (OR = 1.861, 95% CI 1.342–2.581). In the analysis stratified by study quality, both low-quality and high-quality group showed that night shift work was associated with high risk of colorectal cancer (OR = 1.533, 95%CI 1.237–1.899; OR = 1.274, 95% CI 1.065–1.523, respectively).

### Dose-response meta-analysis

**Figure 3:**
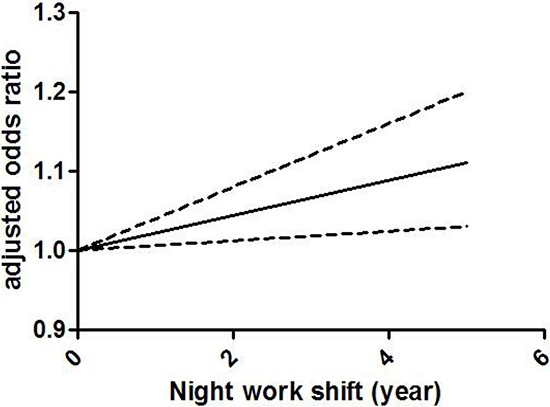
Odds ratio for colorectal cancer by years of night shift work based on the results of the dose-response meta-analyses Solid line represents the estimated odds ratios, while the dotted lines represent the 95% confidence intervals.

Figure 3 demonstrates the dose-response correlation between the night shift work and the risk of colorectal cancer. For 5 years increased in night shift work, the rate of colorectal cancer increased by 11% (OR =1.11, 95% CI 1.03–1.20). Furthermore, the goodness-of-fittest indicated that no statistically significant heterogeneity was found (*p* = 0.27).

### Evaluation of heterogeneity

**Figure 4:**
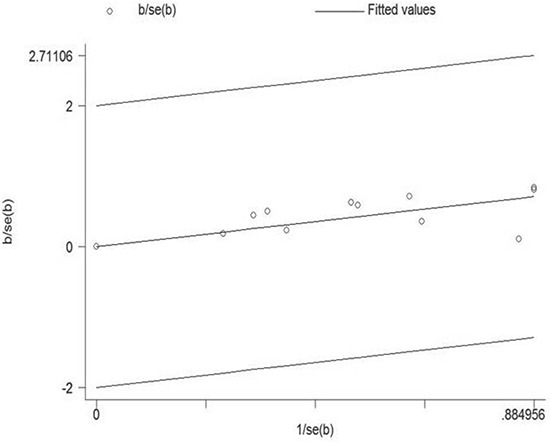
Galbraith plot analysis was used to evaluate heterogeneity It indicated that none of the included studies could be the possible source of heterogeneity.

Because a statistically significant heterogeneity was found among the included studies (I^2^ = 77.7%, *p* < 0.01), the Galbraith plot test was carried out to explore the potential source of heterogeneity from the included studies. However, the result indicated that there was not a specific study could be the major source of heterogeneity (Figure 4). Therefore, meta-regression was used to evaluate the possible source of heterogeneity from the variables in every study. As a result, except gender, all the other variables were considered as the potential sources of heterogeneity in our analysis (Table 2).

**Table 2:** Stratified pooled odds ratio (OR) and 95% confidence intervals (CIs) for the correlation between night work shift and risk of colorectal cancer

Subgroup	Number of studies	OR (95% CI)	Heterogeneity		P for interaction
			*P*	*I^2^* (%)	
Type of Cancer					
Colon cancer	4	1.281 (0.949–1.733)	0.004	74.4	0.005
Rectal cancer	4	1.318(0.814–2.064)	0.003	74.7	
Study design					
Cohort	3	1.318 (0.957–1.219)	0.138	38.2	0.002
Case-control	3	1.630 (1.324–2.007)	0.067	58.2	
Gender					
Male	2	1.328 (1.039–1.697)	0.000	87.8	0.010
Female	4	1.303 (1.100–1.544)	0.318	14.9	
Region					
Europe	3	1.120 (0.959–1.307)	0.021	59.6	0.006
America	3	1.610 (1.293–2.006)	0.064	58.7	
Exposure assessment					
Questionnaire	1	1.440 (1.100–1.890)	-	-	0.002
Interview	4	1.290 (1.073–1.551)	0.000	82.7	
Database	1	1.491 (0.854–2.604)	0.000	77.7	
Control factors					
>3	5	1.248 (1.058–1.472)	0.537	0.0	0.002
≤3	2	1.861 (1.342–2.581)	0.000	79.9	
Study quality					
High	4	1.274 (1.065–1.523)	0.848	0.0	0.004
Low	2	1.533 (1.237–1.899)	0.000	80.9	

### Cumulative meta-analysis

**Figure 5:**
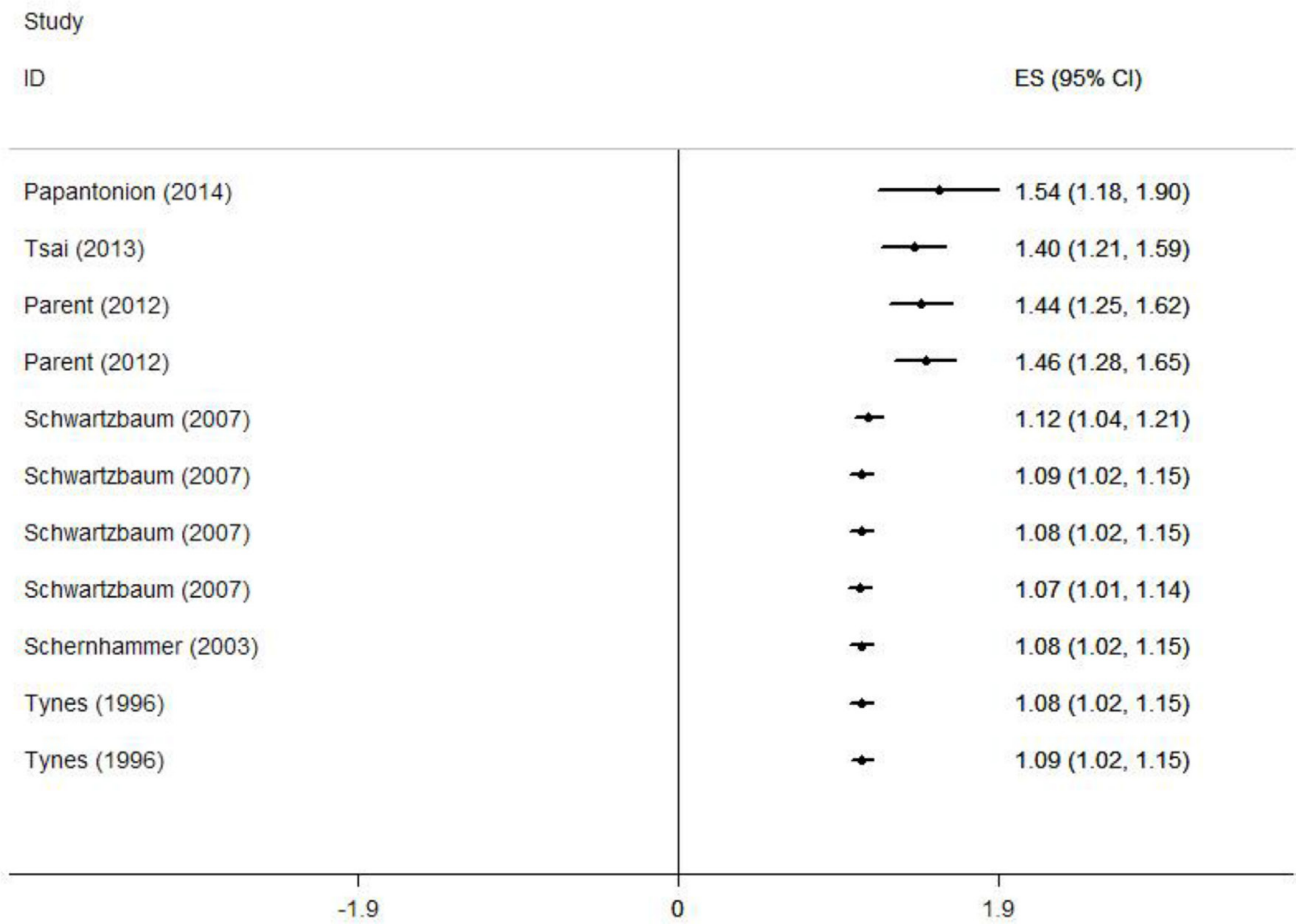
Results from cumulative meta-analysis of the relation between night shift work and risk of colorectal cancer The circles and horizontal lines illustrated the accumulation of estimates as results from each study were added and the 95% confidence intervals became narrower with the increasing sample size, implying that the accuracy of the estimates was progressively increasing by the continuous addition of studies.

Cumulative meta-analysis was carried out by ordering the included studies based on publication year. The result of cumulative meta-analysis indicated the correlation between night shift work and risk of colorectal cancer in chronologic order (Figure 5). The 95% CIs have become narrower with increased sample size, indicating that the accuracy of the estimates was increasing by the continuous inclusion of studies.

### Sensitivity analysis

**Figure 6:**
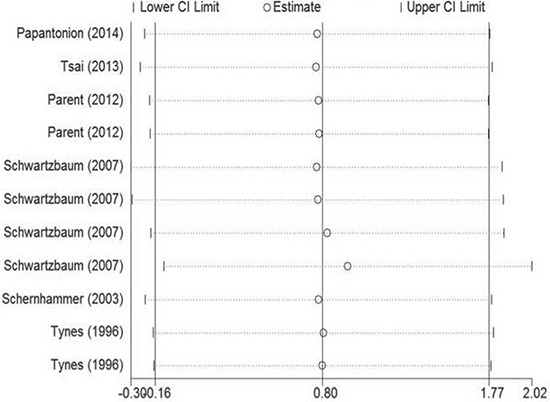
Sensitivity analysis was conducted to evaluate the effect of each study on the overall estimate by sequentially excluding one study in one turn No study could probably affect the summary of risk estimate in this study.

Sensitivity analysis was carried out to assess the effect of one study on the overall estimates by sequentially excluding each study in one turn. In our analysis, no study could possibly affect the pooled risk estimate (Figure 6).

### Publication bias

**Figure 7:**
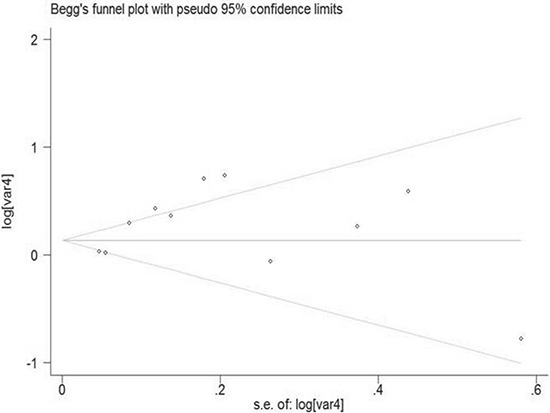
Funnel plot of night shift work and risk of colorectal cancer

**Figure 8:**
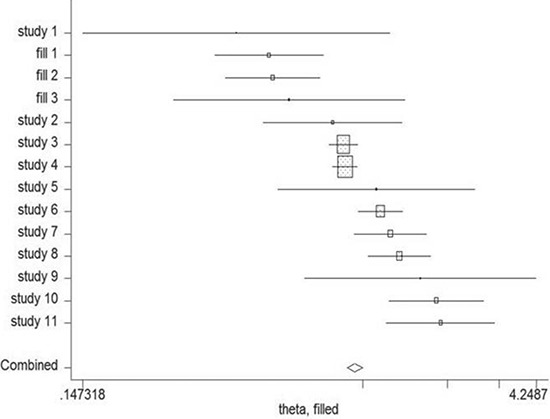
The trim-and-fill test identified 3 possible missing studies

Begg's and Egger's test was carried out to assess the publication bias of the included studies (Figure 7). No evidence of publication bias was detected by either way (*p* = 0.876, *p* = 0.962, respectively). Three possible missing studies were identified by the trim-and-fill test (Figure 8). Nevertheless, all the studies would not change the trend of our results (OR = 1.107, 95% CI 1.046–1.172).

## DISCUSSION

This meta-analysis summarized the results of 6 epidemiologic studies including 3 cohort studies and 3 case-control ones. To the best of our knowledge, it is the first meta-analysis evaluating the correlation between night shift work and risk of colorectal cancer. We found that night shift work was correlated with an increased risk of colorectal cancer (OR = 1.318, 95% CI 1.121–1.551).

A dose-response analysis demonstrated that an increase in night shift work of 5 years was associated with an 11% increase in the risk of colorectal cancer in the population. Similar result was observed in a previous meta-analysis regarding the correlation between night shift work and risk of breast cancer, and a 13% increased risk of breast cancer was found by every 500 nights increase of shift work (RR = 1.13, 95% CI 1.07–1.21) [9]. Therefore, the above results illustrated that an increased risk of colorectal and breast cancer was observed along with the cumulative time of night shift work, indicating that night shift work could probably higher the risk of cancer in a dose-response way.

Quantified Q test and I^2^ test were applied to assess the extent of heterogeneity among the included studies. A statistically significant heterogeneity was found among the included studies (I^2^ = 77.7%, *p* < 0.01). Through the Galbraith plot test, we found that there was not a specific study could be the major source of heterogeneity. It meant that every included study contribute to the heterogeneity of this meta-analysis. We further conducted meta-regression analysis to evaluate the possible source of heterogeneity from the variables in every study. We found that type of cancer, gender, region, exposure assessment, study design, control factors, and study quality were identified as the possible sources of heterogeneity. Therefore, subgroup analysis was performed to analyze the possible source of heterogeneity and evaluate that whether such heterogeneity could be avoided. We detected a significant heterogeneity in the case-control group (I^2^ = 58.2%, *p* = 0.067). On the contrary, a moderate heterogeneity was found in the cohort group (I^2^ = 38.2%, *p* = 0.138), which suggested that cohort study should be a better choice in future studies to avoid potential heterogeneity. Furthermore, we found that heterogeneity could be avoided in the group adjusted for >3 control factors (I^2^ = 0.0%, *p* = 0.537) and the high-quality group (I^2^ = 0.0%, *p* = 0.848), indicating that adjusting for more control factors and recruiting high-quality researches were effective ways to avoid heterogeneity.

Several important molecular signal pathways involving in carcinogenesis are controlled by the circadian timing system [18]. The classic opinion that a unique pacemaker controls all circadian rhythms in physiology and behavior has been outdated [19]. The central clock which resides in the suprachiasmatic nucleus synchronizes numerous peripheral oscillators to maintain coordinated physiology in the internal environment. The peripheral oscillators also connect and communicate to the suprachiasmatic nucleus in turn. The central clock synchronizes the peripheral oscillators in organs far away from the brain through both direct and indirect pathways [20]. In the indirect pathway, peripheral oscillators are controlled by activity-rest cycle and thus, feeding-starving cycle. Most peripheral clocks are influenced by food metabolites including glucose and hormones related to feeding and starving [21]. Besides, body temperature rhythms are also influenced by activity-rest cycle, and it can take part in the synchronization of peripheral oscillators [22]. In the direct pathway, the suprachiasmatic nucleus synchronizes the peripheral oscillators via circulating secreted hormones and neuronal signals [23, 24]. Additionally, body temperature rhythms can also be controlled by suprachiasmatic nucleus directly to influence the synchronization of peripheral oscillators [22, 25].

Long-term exposure to light-at-night and night shift work could disturb the human normal day-night rhythm and lead to circadian disruption. As a result, the internal pacemaker of the synchronization nuclei would be reset, and the output of melatonin would be suppressed [26, 27]. Melatonin illustrated a protective effect against cancer via apoptosis, anti-angiogenesis, anti-oxidation and regulation of the immune system [28]. Apoptosis induced by melatonin in colorectal cancer was mainly based on the nuclear import of HDAC4 and subsequent H3 deacetylation by the inactivation of CaMKIIα [29]. Serum cortisol is another important output of the suprachiasmatic nucleus. A significant correlation was observed between serum levels of TGF-A and IL-6, circadian patterns and serum cortisol in patients with metastatic colorectal cancer [30]. Such interaction between cytokine signaling pathways, serum cortisol, and efferent pathways of the suprachiasmatic nucleus, provide a new perspective for interventions of colorectal cancer patients. Additionally, long-term night work could reduce the exposure of sun light and subsequently decrease the level of vitamin D [31]. A significant inverse correlation between circulating concentration of 25-hydroxyvitamin D [25 (OH) D] levels and risk of colorectal cancer was detected [32, 33]. The risk of some hormone dependent cancers such as prostate and breast cancer are correlated with disrupted circadian rhythms, and dysregulated circulating sex hormones resulted from a disrupted circadian rhythm will lead to an increased risk of those cancers. We found a significant positive correlation between night shift work and risk of colorectal cancer in both female and male groups (OR = 1.303, 95% CI 1.100–1.544; OR =1.328, 95% CI 1.039–1.697, respectively). Considering the different hormone level in male and female, it indicated that circulating sex hormones may not be the main reason for the increased risk of colorectal cancer. In the future, more experimental researches should be conducted to further elucidate the possible molecular mechanism of disrupted circadian rhythms with carcinogenesis in the colorectum.

However, our study has several limitations. Firstly, the definition of period time regarding night shift did not reach a consensus among different studies. Some of studies defined it as work full time in permanent night shifts for more than 1 year [11, 12], some defined it as work on night shift for more than 3 times per month and at least for 1–14 years [15], some defined it as work on night shift for at least 1–5 years [13], while others did not have such requirement but those who ever had worked night shift [14, 16]. Lack of standard definition of night shift in different studies could result in a certain extent of mis-classification, and subsequently could lead to a dilution of summarized estimates when doing analysis. Secondly, though no publication bias was found in our meta-analysis by either Egger's or Begg's test, the selection strategy of published studies could bring about potential publication bias to affect our ultimate findings. Thirdly, considering the heterogeneity in our study, it was inappropriate to choose one single pooled effect to summarize the data, and the results in our meta-analysis should be treated with caution. Therefore, we conducted subgroup analysis to explain the possible sources of heterogeneity. Additionally, half of the recruited articles in our study were case-control studies, which could probably bring about selection and recall bias. Fourthly, based on the characteristics of cohort study and case-control study, the former is a better method to elucidate the correlation between night shift work and risk of colorectal cancer. And the result from cohort study is more credible and valuable since case-control study is more susceptible to confounding factors. Nevertheless, we failed to find a positive relationship in the cohort group compared with the case-control group. The reasons were listed below. 1. Because the NOS score indicated that the included cohort studies were not well designed (with a mean of 7.3), the pooled OR should be treated with caution. 2. The number of cohort study was limited in this meta-analysis, which could bring a negative impact on the result. Therefore, more multi-center, large-sample and well designed cohort studies are of great necessity to better illuminate the relationship between night shift work and risk of colorectal cancer in the future. Finally, a meta-analysis is unable to solve problems with confounding factors in the recruited studies. Inadequate control of known confounding factors could lead to possible bias in direction to either underestimation or exaggeration of the pooled estimates. In our study, 2 articles failed to adjust for three or more than three control factors, which was a main reason for heterogeneity in our study.

In conclusion, this meta-analysis indicated that night shift work was associated with an increased risk of colorectal cancer. Further researches should be conducted to confirm our findings and clarify the potential biological mechanisms.

## MATERIALS AND METHODS

### Literature search

In order to get an overview on night shift work and risk of colorectal cancer, a comprehensive and systematic searching strategy was conducted. We searched for publications up to March 2015 using PubMed, Web of Science, Cochrane Library, EMBASE and the Chinese National Knowledge Infrastructure databases. (Scheduling or night shift or shift work), (colorectal cancer or colon cancer or rectal cancer) and (risk or cancer risk or cancer mortality) were used as key words to select publications. We evaluated all possible articles by checking titles and abstracts, and those meeting the eligibility criteria were retrieved. In addition, other relevant publications were retrieved by evaluating the references of the included articles or reviews regarding the relationship between night shift work and risk of colorectal cancer. This study was planned and performed in accordance to the quality standards for meta-analysis [34, 35]. A total of 79 articles were detected when the above key words were used. After a closer examination, 74 articles were excluded in accordance to titles and abstracts, while 1 article was recruited from the references of the included articles or reviews regarding the relationship between night shift work and risk of cancer [16].

### Inclusion and exclusion criteria

Every included articles were assessed that whether the following criteria were met: (1) cohort or case-control study of the possible relationship between night shift work and risk of colorectal cancer; (2) exact data extracted from every study group; (3) articles published before March 2015 written in English or Chinese; (4) results involving odds ratio (OR) or relative risk (RR), or providing sufficient data to calculate them. If the same population was chosen by more than one study, the one with the largest number of cases or the latest research period was qualified for inclusion. Studies with overlapping data or insufficient information were excluded.

### Data extraction

Information extracted from the studies included name of first author, year of publication, study design, country, sample size, assessment of exposure, adjusted effect estimates for night shift work, and adjusted or matched variables in each study. Considering colorectal cancer is a relatively rare disease, RR was accepted the same as OR. Thus, we used pooled OR to evaluate the possible correlation between night shift work and risk of colorectal cancer. Investigators were divided into two groups to extracted statistics independently from all possibly eligible studies in case of mistakes or omissions. We chose either group discussion or consulting to a third party to resolve any discrepancy.

### Quality evaluation

The quality of each study was assessed via the Newcastle-Ottawa Quality Assessment Scale (NOS) (http://www.ohri.ca/programs/clinical_epidemiology/oxford.asp) by the two groups of authors. Any disagreement was discussed via re-evaluation of study with a third authorized party. The NOS is an eight-item form which allows for evaluating population selection, study comparability, and exposure. Interpretation of the scale is conducted by marking stars for high-quality elements. The number of stars is summed up and used to evaluate the quality of each study quantitatively. The score scale is 0–9. Score <7 was designated to be low-quality, otherwise to be high-quality study.

### Statistical analysis

OR and 95% CI were used to assess the degree of the correlation between night shift work and risk of colorectal cancer. We used the random-effect model with the DerSimonian and Laird method to provide summarized estimation of the relationship between night shift work and risk of colorectal cancer when heterogeneity was found [36], otherwise, we chose the fixed-effect model with the Mantel-Haenszel method for summarizing [37]. Subgroup analysis was performed on study design, country, gender, assessment of exposure and the number of control factors. Quantified Q test and I^2^ test were applied to assess the extent of heterogeneity among the included studies [36, 38], and significant heterogeneity was set at the level of *p* < 0.10. Furthermore, the result of I^2^ test was also used to evaluate the degree of heterogeneity (I^2^ > 50%: large heterogeneity; I^2^ = 25–50%: moderate heterogeneity; I^2^ < 25%:no heterogeneity). Moreover, we also carried out the meta-regression analysis and Galbraith plot test to explain the possible sources of heterogeneity [39]. And a repeated analysis was conducted after excluding studies which probably causing heterogeneity if necessary.

For dose-response meta-analysis, we retrieved studies that included at least 3 levels of night shift work and supplied exact number of case and control groups in each category. The risk estimates were re-summarized via the method proposed by Greenland and Longnecker and Orsini et al [40, 41]. Additionally, the midpoint of the lower and upper boundary in each category was assigned as the average time/frequency of night shift work. If the lower boundary of the lowest category or the upper boundary of the highest category was not provided, the scale of the interval was supposed the same as the preceding category.

Sensitive analysis was performed to assess the effect of one study on the overall result. Cumulative analysis was conducted via ordering studies in accordance to publication year. We used Egger's and Begg's test to evaluate publication bias [42, 43]. Besides, trim-and-fill test was also carried out to evaluate potential publication bias [44]. Trim-and-fill test suggests that the effect sizes of all studies distribute normally around a central point of a graph. If asymmetry is detected, then it adjusts for the possible effect that unpublished studies could have on the summarized result.

All statistical analyses were performed via STATA version 11(StataCorp, College Station, TX., USA).
